# Editorial: Proceedings of ASPL2019 - 8th Asian-Oceanian Symposium on Plant Lipids

**DOI:** 10.3389/fpls.2020.617094

**Published:** 2020-12-08

**Authors:** Xue-Rong Zhou, Ikuo Nishida, Mi Chung Suh, Thomas Vanhercke

**Affiliations:** ^1^CSIRO Agriculture and Food, Canberra, ACT, Australia; ^2^Division of Life Science, The Graduate School of Science and Engineering, Saitama University, Saitama, Japan; ^3^Department of Life Science, Sogang University, Seoul, South Korea

**Keywords:** ASPL2019, lipid synthesis and storage, lipid functions, lipid production, lipid platform

The 8th Asian-Oceanian Symposium on Plant Lipids (ASPL2019) was held in Canberra, Australia, on November 19-22, 2019, accommodated 55 oral talks and 24 posters. ASPL2019 covered a broad range of both fundamental and applied Research Topics related to plant and algal lipid metabolism including lipidomic and lipid metabolic analysis; membrane lipids and environment; wax, sphingolipids, sterols and isoprenoids; lipid droplets and storage lipids; algal and microbial lipids; lipids for food and nutrition; lipase and lipid catabolism; lipids and plant biotechnology; synthetic biology. A Research Topic from Frontiers in Plant Science was open to participants of ASPL2019 to contribute. This Research Topic presents 14 peer reviewed articles contributed by 113 authors.

## Lipid Synthesis and Storage

WRINKLED1 (WRI1) transcription factor plays a central role in the transcriptional regulation of plant fatty acid biosynthesis in seed. Kong et al. summarized the recent understanding of the regulatory mechanism of WRI1 in plant oil biosynthesis and other regulators controlling the expression of *WRI1*. These recent advances provide novel approaches for engineering oil production in plants.

Chlorophyll biosynthesis is important for maintaining high photosynthetic productivity in crops. Wang et al. identified a wheat mutant defective in chlorophyll biosynthesis, displaying an altered leaf color. The mutant has a single G664A substitution in the gene *TaCHLI-7A* encoding the subunit I of the Mg-chelatase, and the resultant D221N substitution in the mutant protein abolishes an interaction with the normal TaCHLI-7A protein, which is required for Mg-chelatase function. Interestingly, the mutant also displayed reduced FA content in seed and leaf. Thus, the mutant may serve as a useful resource for a better understanding chlorophyll and lipid biosynthetic pathways in common wheat.

In recent years, advances in plant lipid metabolic engineering have led to transgenic strategies that allow for the storage lipid triacylglycerol (TAG) to accumulate in non-seed tissues of tobacco, sorghum, and sugarcane. Xu, Vanhercke et al. overexpressed WRI1, diacylglycerol acyltransferase 1 (DGAT1), and Oleosin oil body protein in potato leaves. The combined “Push, Pull, Protect” strategy resulted in a 30-fold increase in TAG levels. The authors observed an inverse correlation between lipid and starch content, suggesting a redistribution of carbon toward TAG as a new sink. Interestingly, transgenic mesophyll chloroplasts contained a large number of plastoglobuli, hinting at phytol esters competing with TAG for photosynthetic carbon. The second paper also by Xu, Akbar et al. explored the effect of downregulating TAG turnover and inhibition of starch biosynthesis in a previously established transgenic potato line that accumulates TAG levels in tubers. To this end, the SDP1 lipase and AGPase were downregulated *via* RNA interference (RNAi) to further optimize the flux of storage carbon toward lipids. Super-transformed events accumulated up to 7% TAG in transgenic tubers. In addition, the authors observed increased levels of polar membrane lipids, as well as significant changes in starch granule composition, morphology, and functionality.

Erucic acid is a valuable industrial fatty acid with many applications for biodegradable and environmentally safe oil products such as biodiesel, lubricants, surfactants, pharmaceuticals, cosmetics, soaps, rubber, and nylon. There are low- and high-erucic acid accessions of *Brassica napus*. Lu et al. examined the distribution of erucic acid-containing lipids and the gene transcripts encoding the enzymes involved in pathways for its synthesis and incorporation into triacylglycerols in seeds. This is also another excellent example of application of matrix assisted laser desorption/ionization-mass spectrometry imaging, linked to the molecular mechanism.

Oil bodies and lipid droplets are the major lipid particles of neutral lipids assembling, storing, and suppling in plants. These particles and the oil body proteins are also involved in many other cellular processes. Shao et al. reviewed recent work on the structural or metabolic roles of these proteins in oil body biogenesis, stabilization and degradation, lipid homeostasis and mobilization, hormone signal transduction, stress defenses, and various aspects of plant growth and development. The review suggested opportunities for increasing stress resistance, enhancing plant yield or increasing the total TAG content in plant for a variety of industrial applications.

In addition to seed oil storage, silique dehiscence plays another important role in seed oil production. Silique dehiscence enables mature seeds to be released from plants, thus its timing and degree affect the harvest and yield. The review article contributed by Yu Y.-K. et al. summarizes the recent understanding of the mechanism and regulation of silique dehiscence, especially the genetic elements controlling silique dehiscence and their silique dehiscence.

## Physiological Functions of Plant Lipids

Lipidomics is an emerging technology that allows us to globally characterize and quantify lipids within biological matrices including biofluids, cells, whole organs, and tissues. Yu D. et al. applied this technology in characterizing the changes in individual lipid molecular species among salinity-stress-treated barley cultivars displaying different salinity tolerance. They found a general decrease in most of the galactolipids in plastid membranes, and an increase of glycerophospholipids and acylated steryl glycosides in extraplastidial membranes in barley roots. They also revealed salinity-induced oxidative damages to plastidial and extraplastidial membrane lipids, which are associated with sensitivity to salinity stress in barley.

Terrestrial crop productivity is significantly reduced by drought. The aerial organs of land plants increase total loads of cuticular waxes to decrease non-stomatal water loss under drought. Yang et al. revealed that an AP2/DREB-type transcription factor RAP2.4, which is induced by ABA-, drought-, osmotic-, and salt-stress upregulates the expression of *KCS2* and *CER1*, which are required for the production of alkanes, and thereby, contributes to an increase in total wax loads in *Arabidopsis* leaves. This study shows that RAP2.4 might be applicable in the production of alkanes and the development of crops with enhanced drought tolerance.

## Lipid Production

The long-chain omega-3 polyunsaturated fatty acids such as EPA and DHA are essential components of animal cell membranes and important contributors to human health. However, increased demand for these fatty acids has led to overfishing of many source species. Petrie et al. developed a novel form of canola, containing fish oil-levels of DHA in its seed oil, the first such crop to obtain regulatory approval in any jurisdiction, with a significant decrease of the ω6:ω3 ratio. Multiple field trials have confirmed the crop to be a reliable land-based source of these essential fatty acids.

Nutritional value and health benefits of peanut seed oil, which contains 2.5 to 8.5% very long chain saturated fatty acids (VLCSFAs) can be enhanced by the reduction of VLCSFA levels. The first step of very long chain fatty acid (VLCFA) synthesis is processed by 3-ketoacyl-CoA synthase (KCS), which catalyzes the condensation of a C2 unit from malonyl-CoA to acyl-CoA. Among 30 putative *KCS* isoforms identified from the peanut genome, Huai et al. revealed that *AhKCS1* and *AhKCS28*, which are predominantly expressed in the developing seeds and localized in the ER, are the key genes involved in the synthesis of VLCFAs in seeds.

Fatty alcohols are widely used in industrial applications, but their production process is accompanied by a harsh work environment and environmental contamination. It is desirable to find organisms that efficiently produce fatty alcohols. Munawaroh et al. revealed that disruption of *HetN* and *PatS*, the repressors of heterocyst differentiation enhanced accumulation of fatty alcohols in the heterocysts of *Anabaena* sp. PCC 7120 *hglT*, which is a loss-of-function mutation in glycosyltransferase required for the synthesis of heterocyst-specific glycolipids. This result provides a novel strategy to produce fatty alcohols in atmospheric nitrogen-fixing Anabaena, which is a great candidate for bioproduction.

Branched fatty acids are widely used in the petrochemical industry due to their high oxidative stability and low melting temperature. Hydrogenation of cyclopropane fatty acids such as dihydrosterculic acid (DHSA) can convert them into branched fatty acids, thus providing potential feedstock. Okada et al. reported that overexpression of *E. coli* or cotton cyclopropane fatty acid synthases and Arabidopsis DGAT1 silencing endogenous oleoyl desaturase resulted in 15% of DHSA in *Nicotiana benthamiana* leaf TAGs. This study shows a possible route to DHSA production in vegetative tissue.

## New Platform

Traditional genetic studies in plants are both time-consuming and labor-intensive. The need to generate stably transformed plants means that the engineering of multi-gene metabolic pathways quickly becomes daunting. Current transient assays are typically limited to hundreds of genes depending on the lab infrastructure. In an attempt to develop a high throughput screening platform that is amenable to large complex libraries, Pouvreau et al. explored tobacco protoplasts as a new plant synthetic biology chassis to identify regulators of lipid metabolism. The authors show that protoplasts can be successfully engineered to hyper-accumulate storage lipids, and subsequently isolated individually by fluorescence-activated cell sorting.

## Author Contributions

X-RZ, IN, MCS, and TV all contributed to writing this Editorial of the Research Topic on Proceedings of ASPL2019 - 8th Asian-Oceanian Symposium on Plant Lipids edited in 2019-2020. All authors contributed to the article and approved the submitted version.

## Conflict of Interest

The authors declare that the research was conducted in the absence of any commercial or financial relationships that could be construed as a potential conflict of interest.

