# Corrigendum: Editorial: Proceedings of ASPL2019 - 8th Asian-Oceanian Symposium on Plant Lipids

**DOI:** 10.3389/fpls.2021.728770

**Published:** 2021-07-14

**Authors:** Xue-Rong Zhou, Ikuo Nishida, Mi Chung Suh, Thomas Vanhercke

**Affiliations:** ^1^CSIRO Agriculture and Food, Canberra, ACT, Australia; ^2^Division of Life Science, The Graduate School of Science and Engineering, Saitama University, Saitama, Japan; ^3^Department of Life Science, Sogang University, Seoul, South Korea

**Keywords:** ASPL2019, lipid synthesis and storage, lipid functions, lipid production, lipid platform

In the original article's Funding statement, the stated New Breeding Technologies Development Program Project number (“PJ04781022020”) was incorrect. The correct number is “PJ014781022020”.

The authors apologize for this error and state that this does not change the scientific conclusions of the article in any way. The original article has been updated.

